# Expression of CXCL8 (IL-8) in the Pathogenesis of T-Cell Acute Lymphoblastic Leukemia Patients

**DOI:** 10.7759/cureus.45929

**Published:** 2023-09-25

**Authors:** Sandeep Pandey, Ranjana Singh, Nimra Habib, Vivek Singh, Rashmi Kushwaha, Anil K Tripathi, Abbas A Mahdi

**Affiliations:** 1 Biochemistry, King George’s Medical University, Lucknow, IND; 2 Pathology, King George’s Medical University, Lucknow, IND; 3 Clinical Hematology, King George’s Medical University, Lucknow, IND

**Keywords:** cancer pathogenesis, t-cell acute lymphoblastic leukemia, cancer therapeutics, molecular biomarker, interleukin-8

## Abstract

Background

Inflammation plays a very important role in the pathogenesis of a wide range of diseases, such as atherosclerosis myocardial infarction, sepsis, rheumatoid arthritis, and cancer. This study aimed to investigate the association of IL-8 in T-cell acute lymphoblastic leukemia (T-ALL) patients.

Methodology

IL-8 levels were estimated in 52 individuals. Of the study population, 26 were T-ALL patients (all phases of leukemia were included in the study) and 26 were disease-free healthy volunteers. In this study, we employed flow cytometry, enzyme-linked immunosorbent assay, reverse transcription-polymerase chain reaction test, and western blot analysis.

Results

IL-8 was significantly higher in all T-ALL patients than in healthy volunteers. IL-8 levels showed a significant positive correlation in T-ALL patients at the genomic and proteomic levels.

Conclusions

Higher serum IL-8 levels were associated with the advanced disease stage of the clinicopathological parameters. Our results indicate that monitoring IL-8 has a role in modulating disease sensing in T-ALL and may represent a target for innovative diagnostic and therapeutic strategies.

## Introduction

T-cell acute lymphoblastic leukemia (T-ALL) is a blood-related aggressive malignancy that accounts for ~25% of adult and ~15% of childhood malignancies. It arises from the clonal proliferation of bone marrow-derived circulating T lymphocytes that are home to the thymus. It mainly affects children and young adults and is associated with poor disease outcomes. Although generally T-ALL cells remain confined to the thymus, in most cases, leukemia cells can migrate to the bone marrow [1]. The expression of IL-8 has been found to be significantly higher in a wide range of cancers than in normal tissues [2]. Consistent with this, a high concentration of IL-8 has been identified in the serum of cancer patients and is correlated with the progressive size of the tumor, stages, and prognosis [3-6]. It has been recently found that the gene that codes for interleukin-8 (*CXCL8*) is highly expressed in chemorefractory leukemia cells (T-ALL) and has been proposed to play several functions in T-ALL cells [7]. Interleukin-8 (*CXCL8*) is a cytokine with a molecular mass of 8.4 KDa and is a specified chemoattractant for neutrophils. It likely plays an important role in the inflammation associated with a vast range of illnesses, such as myocardial injury, shock, pneumonia, sepsis, rheumatoid arthritis, and respiratory distress syndrome, in newborns [8]. Previous studies have revealed that IL-8 is not produced constitutively but is secreted only in response to specific stimuli, such as lipopolysaccharide stimulation of human monocyte-macrophage cells, endothelial cells, tumor necrosis factor, and IL-1 stimulation of fibroblasts, epithelial cells, synovial cells, and mesothelial cells [7-11]. However, Brennan et al. showed that IL-8 is constitutively produced in the synovial cells of rheumatoid arthritis patients [12] but human B and T cells have not been reported to produce IL-8. Kaashoek et al. [13] and Yoshida et al. [14] found the constitutive production of IL-8 in the human bladder carcinoma cell line 5637 and thyroid carcinoma cell line KHM-5M, respectively. This study aimed to investigate the constitutive production of IL-8 by leukemia (T-ALL) cells.

A preprint of this article was previously published on Research Square (https://www.researchsquare.com/article/rs-1426745/v1).

## Materials and methods

Patient samples

Human blood samples from T-ALL patients (N = 26) and healthy controls (N = 26) were collected in ethylenediaminetetraacetic acid vials using the venipuncture sampling method. Samples were obtained from the Department of Pathology and Biochemistry at King George’s Medical University after informed consent was obtained from all patients enrolled in the study. Demographic data and hematological examination reports of leukemia patients are summarized in Table 1. The ethics committee of King George’s Medical University, Lucknow, approved this study (reference code: 96th ECM II A/P22, dated May 10, 2019). Written informed consent and diagnosis forms were collected from the Department of Pathology, King George’s Medical University, Lucknow.

**Table 1:** Demographic data and hematological examination report of leukemia (T-cell acute lymphoblastic leukemia) patients.

Characteristics of patients	Examination report
Patients/Healthy controls	26/26
Male/Female	36/16
Specimen	Peripheral blood sample
Age at diagnosis, year median (range)	21.375 (4–40)
Hemoglobin	6.8875 g/dL (average)
Total leucocyte count	7.88125 cells/mm^3^ (average)
Red blood cell count	2.158 × 10^12^/L (average)
Blast cells (range)	79.8 (average)
Combination of markers	CD2, CD3, CDE, CD4, CD5, CD7, CD8, CD10, CD13, CD33, CD34, CD38, CD99, CD117, cCD3, and TdT

Sample size calculation

The present study was a case-control study and the sample size was calculated using the following sample size formula: sample size = Z^2^_1-α/2 _p(1-p)/d^2^, where Z^2^1-α/2 is the standard normal variate (at 5% type 1 error (p < 0.05), it is 1.96, and at 1% type 1 error (p < 0.01), it is 2.58). As in most studies, p-values are considered significant at 0.05; hence, 1.96 was used in the formula. The expected proportion in the population was based on previous studies or pilot studies. d is the absolute error or precision which was decided by the researchers.

The prevalence of T-ALL is 2-3% in India according to the Indian Council of Medical Research India report 2017. The sample size was calculated using this report. The sample size required for the study was 52 (including cases and controls).

The clinical diagnosis of T-ALL was based on the patient’s presentation, morphology on a peripheral blood smear, and bone marrow using Leishman staining. Further immunophenotyping of blast cells was performed by flow cytometry using markers such as CD34, CD33, CD14, CD20, CD10, CD19, HLA-DR, TdT, CD2, CD3, CD5, CD7, CD13, CD19, CD20, CD23, CD45, CD64, CD79a, CD117, and CD200. The characteristic features of the patients are presented in Table 1.

**Figure 1:**
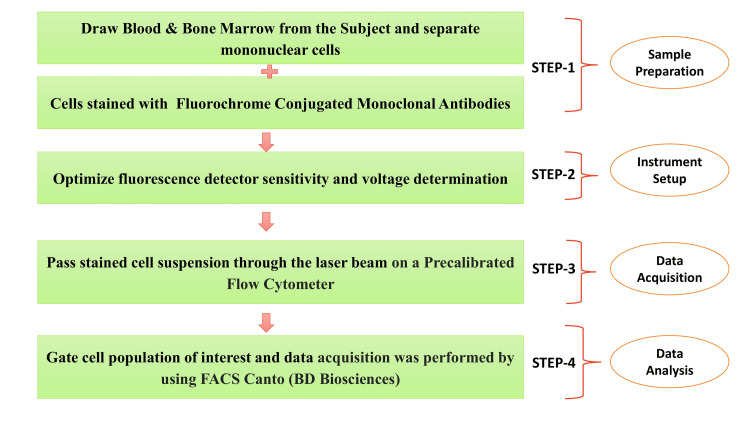
Flowchart of the methodology: flow cytometry.

**Figure 2:**
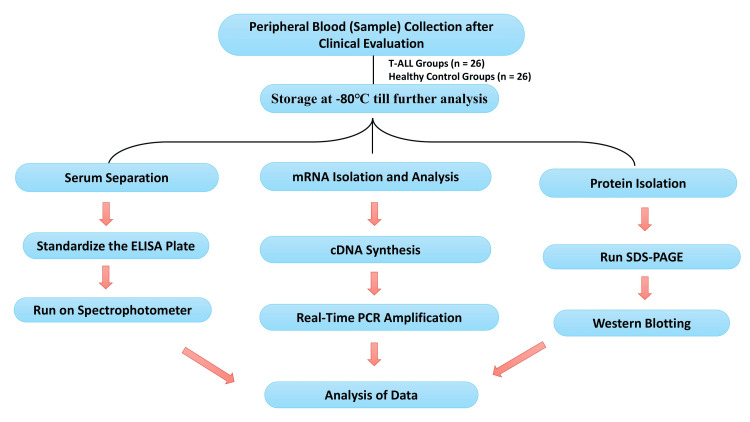
Flowchart of the methodology: enzyme-linked immunosorbent assay, reverse transcription-polymerase chain reaction test, and western blotting.

The written informed consent forms from all patients were obtained before peripheral blood collection. Serum samples from all blood samples were separated by centrifugation and stored at −80°C until further processing and analysis. Serum IL-8 levels were determined by enzyme immunoassay (EIA) using commercially available kits from Abcam, according to the manufacturer’s instructions and protocol. The detailed characteristics of T-ALL patients, including clinical and histopathological reports, were collected from case files maintained at the Department of Pathology, King George’s Medical University, Lucknow. Most experiments were performed immediately after obtaining samples in the Department of Biochemistry. The flowchart for the complete methodology of this study is systematically described in Figure 1 and Figure 2.

Peripheral blood smear and bone marrow staining

Leishman dye was prepared in methanol (0.2 g of Leishman powder and dissolved in 100 mL of methanol). After preparing blood films, they were allowed to air dry. The slides were flooded with Leishman stain for two minutes and washed in a stream of buffered water for two minutes to acquire a pinkish tinge. In cases of suspicion of leukemia, blood films made from buffy coat preparations were stained with Leishman’s stain.

Blast cell conformation by flow cytometry

The combinations of monoclonal antibodies used were labeled with fluorescein isothiocyanate (FITC), phycoerythrin (PE), pyridinyl chlorophyllin (PerCP), and phycoerythrin-Cy7 (PE-Cy7). Anti-CD45 V500-A, anti-CD34 PercP-A, anti-CD33 APC-A, anti-CD79a, APC-A, anti-CD13-PE-A, anti-CD19-PE, anti-CD25-FITC-A, anti-CD20-V450-A, anti-CD7-FITC-A, anti-CD10-PE, anti-CD19-PE, anti-CD20-V450-A, anti-CD14-APC, anti-CD20-V450-A, anti-CD14-APC, anti-CD-64 FITC-A, anti-CD117-PE, human leukocyte antigen (HLA)-DR-APC, anti-CD14-APC, and many more antibodies were procured from BD Biosciences (San Jose, California, USA). We followed the stain lyse wash method; the appropriate number of FACS tubes was labeled for the name of the patient and the combination of fluorochrome-conjugated monoclonal antibodies. In each tube, 100 μL of the sample (whole blood) was pipetted into the tube and poured with 20 μL of antibody/antibody cocktail in the respective tubes, which were incubated in the dark for 10-15 minutes. After incubation, 2 mL of diluted FACS lysis solution was added to each tube. The samples were centrifuged at 200-300 g for three to five minutes. The supernatant was discarded, the pellet formed at the bottom of the tube was broken, and the remaining cells were washed two to three times with sheath fluid. The cells were resuspended again in approximately 0.5 mL of sheath fluid and run on a precalibrated flow cytometer. Data acquisition was performed using FACSCanto (BD Biosciences, San Jose, California, USA).

Reverse transcription-polymerase chain reaction

Total mRNA from blood cells was isolated following the TRIzol method. The concentration of mRNA and its structural integrity were confirmed using a Nanodrop spectrophotometer from Thermo Scientific (2000 UV-Vis). Only RNA with ratios ranging from 1.9 to 2.0 absorbance at 260/280 nm was used. According to the protocol, the isolated mRNA was reverse-transcribed using the high-capacity cDNA reverse transcription kit (4368814). Quantitative reverse transcription-PCR (qRT-PCR) was performed using PowerUp SYBR green master mix (ABI-A25741) on a 7500 Fast Real-Time PCR system (Applied Biosystem, Thermo Scientific). Quantification was performed with the ΔΔ Ct method with β-actin serving as a reference gene, and the RT-PCR results were analyzed using DataAssist software (Thermo Scientific). The sequences of the primers for IL-8 and β-actin are summarized in Table 2.

**Table 2:** Sequence of the oligonucleotide primers used in real-time polymerase chain reaction for IL-8 and β-actin detection.

Gene	Primer sequence	bp
Interleukin-8	(F) ATGACTTCCAAGCTGGCCGTG (R) TGAATTCTCAGCCCTCTTCAAAAACTTCTC	297
Beta-actin	(F) TGACGGGGTCACCCACACTGTGCCCATCTA (R) CTAGAAGCATTGCGGTGGACGATGGAGGG	661

All primers were analyzed using positive controls by executing melting profiles following qRT-PCR, and the sizes of products were checked by gel electrophoresis (2.2% agarose). The conditions for performing PCR were as follows: 40 cycles of 15 seconds at 95°C, 15 seconds at annealing temperature (60°C for all other genes), and 15 seconds at 72°C. Exemplars were investigated in duplicate for a minimum of three sets of individual experiments as indicated.

Western blotting

Blood cells were harvested, the proteins were isolated by RIPA lysis buffer, and the protein concentration was measured by the BCA method (bicinchoninic acid assay) with a spectrophotometer from Thermo Scientific at 562 nm. Proteins were separated by 15% SDS-polyacrylamide gel electrophoresis (SDS-PAGE) and transferred onto PVDF membranes (Bio-Rad). Protein was incubated overnight at 4°C with primary antibodies to form immune complexes. Blots were washed two to three times properly and incubated with HRP-conjugated secondary antibodies for one hour. Bands of the immunoreactive protein were visualized and identified with the help of a scanning system machine named Odyssey LI-COR.

Statistical analysis

All data were analyzed using GraphPad Prism-9 (GraphPad Software, Boston, MA, USA) and SPSS version 16.0 (IBM Corp., Armonk, New York, USA). We performed the Student’s t-test and receiver operating characteristic (ROC) curve analysis (Metaboanalyst version 5.0). All comparisons were made relative to healthy controls (non-cancerous), and the significance of the difference was indicated as *p < 0.01, **p < 0.001, ***p < 0.0001, and ****p < 0.00001. All quantitative data are presented as the mean ± SD from a minimum of three samples per data point.

## Results

**Figure 3:**
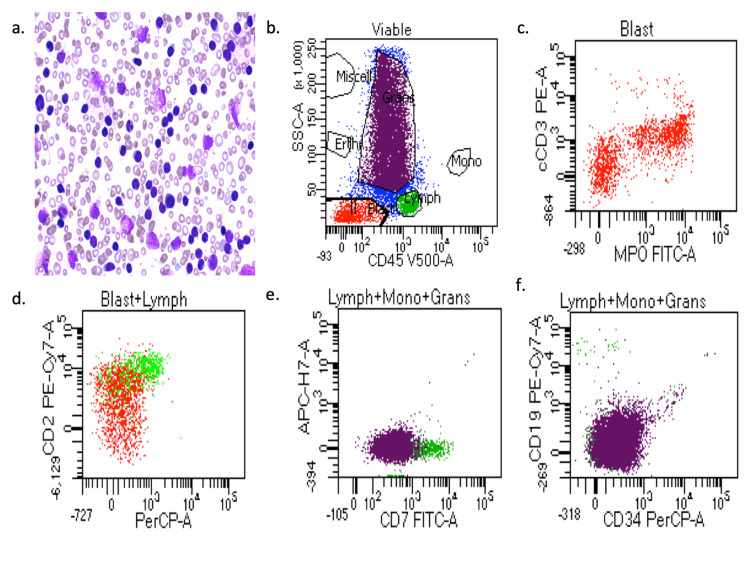
Clinical diagnosis results confirmed by flow cytometry on peripheral blood smear PBS and bone marrow aspiration. (a) Representative images of Leishman staining of the blood of T-cell acute lymphoblastic leukemia (T-ALL) patients. (b-f) Flow cytometry results show all kinds of cells in T-ALL patients, which were plotted on CD45 with side scatter and plotted on the marker and marker versus marker for the confirmation of T-ALL.

In this study, we included the peripheral blood and bone marrow aspirates of T-ALL patients. The morphology of blast crisis cells from peripheral blood smears, aspirates from the smears of bone marrow, and bone marrow trephine biopsy specimens, along with immunophenotypic findings and flow cytometry, are shown in Figure 3a and Figure 3b, which shows that blast cells were stained with Leishman dye. Furthermore, flow cytometry results helped identify phenotypes with different biomarkers, such as CD34, CD33, CD14, CD20, CD10, CD19, HLA-DR, TdT, CD2, CD3, CD5, CD7, CD13, CD19, CD20, CD23, CD45, CD64, CD79a, CD117, and CD200. Blast cells were gated on CD45 versus side scatter. The expression of myeloid markers (CD13, CD33, CD117, CD14, CD64, and cMPO), B-lymphoid markers (CD19, CD20, CD79a, and CD10), T-lymphoid markers (CD3, cytoplasmic CD3, CD2, CD5, and CD7), and immaturity markers (CD34, TdT, and HLA-DR) was assessed. We performed flow cytometry analysis of different patients, as shown in Figures 3c-3e. The results are summarized in Table 1. Overall, 90% of blast cells were gated on dim CD45 and extended to the monocytic region on CD45 versus the side scatter plot, as shown in Figure 3f. A reliable number of CD45 events (20,000) were measured, and we found that 95% of patients had approximately 15,000 blast cells in 20,000 events. Flow cytometry data confirmed the presence of blast cells in T-ALL patients.

Molecular study

**Figure 4:**
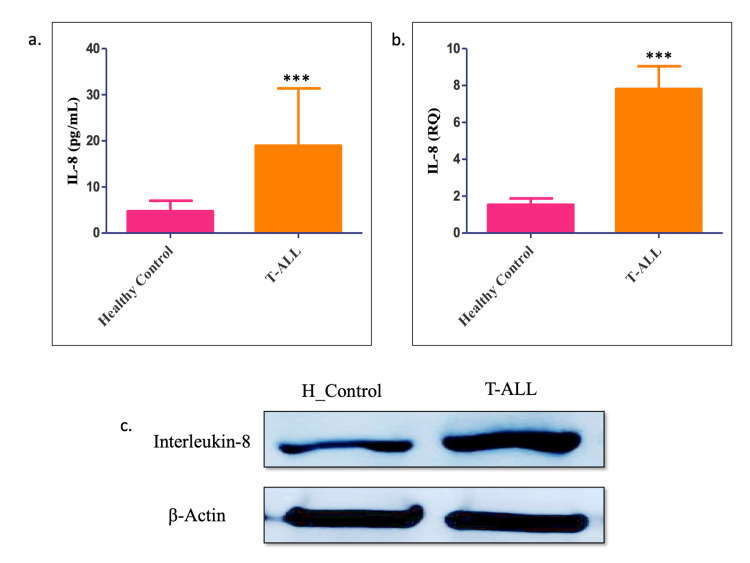
IL-8 expression measured by enzyme-linked immunosorbent assay, reverse transcription-polymerase chain reaction test, and western blotting in T-cell acute lymphoblastic leukemia (T-ALL). (a-c) The IL-8 expression level was estimated in T-ALL patients at the serum, genomic, and proteomic levels. All results follow the same trajectory. All quantitative data are the mean ± SD, **** p < 0.00001, Student’s t-test (paired).

The expression levels of serum IL-8 were elevated in all T-ALL patients (19 ± 2.3, p < 0.0001) compared to healthy individuals (4.8 ± 2.2). IL-8 levels at the serum (ELISA), genomic (RT-PCR), and proteomic levels (western blot) in T-ALL (RQ = 7.8 ± 1.2 p < 0.0001) patients were higher in different histopathological subgroups than in healthy individuals (1.542 ± 03342), as shown in Figure 4.

**Figure 5:**
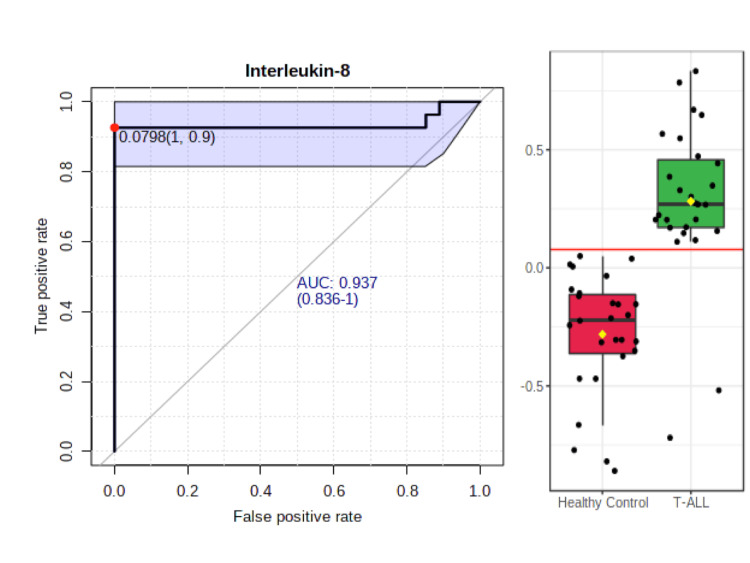
Receiver operating characteristic curve for IL-8 in T-cell acute lymphoblastic leukemia (T-ALL). The area under the receiver operating characteristic analysis showed that IL-8 (area under the curve (AUC) = 0.937; 0.836-1) had a cut-off value (0.0798; 1, 0.9).

The ROC curve analysis indicated that both IL-8 showed good discriminative efficiency among the healthy volunteers and all leukemia/T-ALL patients (IL-8: AUC = 0.93553, p = 5.0815e-9). Moreover, the ROC curves, as shown in Figure 5, suggested that IL-8 possesses good sensitivity and specificity to differentiate healthy volunteers from T-ALL patients.

## Discussion

The patients were registered from different age groups in the Department of Clinical Hematology, King George’s Medical University, Lucknow. Physical history, complete blood count, blood chemistry, bone marrow aspiration, biopsy, cytogenetic analysis, and FACS were performed as well and examination reports of these tests were procured from the Department of Pathology, King George’s Medical University, Lucknow. These tests and procedures were used to diagnose the stages and metastasis of T-ALL. Cytogenetic analysis and FACS were confirmatory tests to diagnose T-ALL from other different types of leukemia. Individuals with a history of any other type of malignancy and patients who were already undergoing chemotherapy were excluded from the study. All patients were diagnosed with T-ALL on bone marrow and flow cytometry. Cytogenetic analysis results were confirmed for the study and written informed consent was taken from all patients. The presented results showed the substantial constitutive secretion of IL-8 by T-ALL cells. Previous studies have reported that IL-8 production occurs mostly through a malignant origin, which may contribute to IL-8-mediated processes such as chronic inflammatory disease state. Some studies have explored that the IL-8 expression in T-ALL cells is regulated by the bone marrow microenvironment. The bone marrow microenvironment emerges as a critical player in cancer biology. However, most isolated parts of the bone marrow have been investigated in the case of acute myeloid leukemia (AML), for example, either the mesenchymal stromal cells [15,16] or bone marrow hypoxia [17,18] and their consequences on blasts of AML cells. CXCL8 is a well-identified pro-inflammatory cytokine and a strong chemoattractant for neutrophils [19]. Besides the physiological function, a few environmental stresses such as acidosis, hypoxia, and chemotherapy have also been attributed to the induction of IL-8 in tumor tissue [20]. In addition, constitutionally elevated IL-8 levels have been observed in a vast range of cancers, such as prostate cancer [21], colorectal cancer [22], and non-small cell lung cancer [23]. IL-8 is a key chemokine that performs different biological functions by binding to two of its major receptors, chemokine (CX-C motif) receptors 1 and 2 (CXCR1 and CXCR2) [24]. In our transcriptomic and translational study, we found that IL-8 was highly expressed in T-ALL samples compared to control samples of healthy individuals. IL-8 expression was much higher than that of the other enhanced expressed genes. This suggests that this gene plays an important role in T-ALL blood/tissue samples, making it unique and critical in leukemia development and progression compared to other genes. In recent times, research related to the identification of some novel biomarkers has become increasingly vital for the very early detection of the stages of cancers and the evaluation of chemotherapeutic strategies in personalized medicine [25,26]. Thus, IL-8 is highly expressed in T-ALL patients and may prove to be a good candidate biomarker for early disease detection. To further elucidate IL-8 in the pathogenesis of T-ALL requires a larger sample size and its association with different inflammatory markers.

Limitations of the study

This is a pilot study evidencing the importance of serum IL-8 in predicting the possible role in the pathogenesis of T-ALL. The limitations of our study are that individuals with a history of any other type of malignancy were not included in the study; patients who were already undergoing chemotherapy were excluded from our study; and, finally, the involvement of a limited number of volunteers (both for case and control) in the study. All findings suggest the need for a higher number of patients and multicenter studies in which all these limitations are taken into account.

## Conclusions

Our results are consistent with previous reports demonstrating that IL-8 level was relatively positive in the disease stage and that the increased IL-8 was primarily correlated with the advancement of T-ALL. Thus, IL-8 is considered to play a role in the pathogenesis of T-ALL, and quantification of IL-8 levels in leukemia conditions might be more useful and feasible in the clinical setting for the prediction of drug responses, where it may represent a presumptive target for innovative diagnostic and effective therapeutic approaches. However, further research is needed, including a greater number of patients with T-ALL, and estimating the IL-8 levels in leukemia patients may hold the key to the additional predictive values on the recurrence of the disease and its prognosis.
